# Vestibular Compensation in Unilateral Patients Often Causes Both Gain and Time Constant Asymmetries in the VOR

**DOI:** 10.3389/fncom.2016.00026

**Published:** 2016-03-29

**Authors:** Mina Ranjbaran, Athanasios Katsarkas, Henrietta L. Galiana

**Affiliations:** ^1^Department of Biomedical Engineering, McGill UniversityMontreal, QC, Canada; ^2^Department of Otolaryngology, McGill UniversityMontreal, QC, Canada

**Keywords:** vestibulo-ocular reflex, vestibular compensation, commissural neuron circuitry, unilateral vestibular lesion, plasticity

## Abstract

The vestibulo-ocular reflex (VOR) is essential in our daily life to stabilize retinal images during head movements. Balanced vestibular functionality secures optimal reflex performance which otherwise can be distorted by peripheral vestibular lesions. Luckily, vestibular compensation in different neuronal sites restores VOR function to some extent over time. Studying vestibular compensation gives insight into the possible mechanisms for plasticity in the brain. In this work, novel experimental analysis tools are employed to reevaluate the VOR characteristics following unilateral vestibular lesions and compensation. Our results suggest that following vestibular lesions, asymmetric performance of the VOR is not only limited to its gain. Vestibular compensation also causes asymmetric dynamics, i.e., different time constants for the VOR during leftward or rightward passive head rotation. Potential mechanisms for these experimental observations are provided using simulation studies.

## 1. Introduction

The Vestibulo-ocular reflex is a short latency and involuntary eye movement that is essential to maintain gaze and stabilize vision during head movements. The neural pathway for the VOR is rather simple and includes three main components: sensors, central processing and the oculomotor plant. Angular and linear head perturbations are sensed by the vestibular apparatus (the semicircular canals and the otolith organs) located in the inner ear. Sensory information is relayed through the vestibular afferents to the brainstem centers including the vestibular nuclei (VN) and the Prepositus-Hypoglossi (PH). These centers act as the main controller and combine sensory and motor information to drive the extraocular muscles and move the eyeballs in the appropriate direction. VOR nystagmus consists of two components; slow compensatory eye movements usually in the opposite direction to the head movements and fast re-orienting eye movements usually in the same direction as the head movements. A switching mechanism controls the slow/fast sequences to keep the eyes in their feasible range during such nystagmus. In clinical tests, the VOR is characterized by its gain defined as the ratio of peak eye velocity to peak head velocity during harmonic testing or short pulse perturbations. Normally this gain is close to unity with vision but only ≈0.6–0.8 in the dark (Paige, 1989).

The VOR system has a bilateral (symmetric) neural structure that includes commissural connections between the two sides of the brainstem. The normal function of the VOR relies on balanced reciprocal stimulation of vestibular sensors on both sides. Partial or complete loss of the afferent input from one side due to any lesion, disease or surgery results in an asymmetry in the VOR circuit and its dynamics. Unbalanced sensory projections to the brainstem circuit often cause severe postural and oculomotor disturbances with symptoms of dizziness, nausea, vertigo and exacerbation of symptoms with head movement (Lopez, 2013). Fortunately, many of the symptoms, e.g., spontaneous nystagmus, recover rapidly as vestibular *compensation* takes place (Curthoys, 2000; Paterson et al., 2004). Vestibular compensation is the process that restores some functionality to the vestibular system after vestibular lesions.

Multiple and parallel plastic processes at various sites in the brain are involved in vestibular compensation (see review Dutia, 2010) at the level of cortical, cerebellar and brainstem circuits, and at the level of the periphery (Cullen et al., 2009). In summary, modifications to compensate for vestibular deficits include: (i) adaptive changes in the sensitivity and resting activity of vestibular neurons and the commissural network (Galiana et al., 1984; Graham and Dutia, 2001; Cullen and Minor, 2002) as well as in the response of the peripheral signals (Cullen et al., 2009); (ii) changes in the inhibitory control of the brainstem vestibular network by the cerebellum (Darlington and Smith, 2000); (iii) Neurogenesis and gliosis in the ipsi-lesional VN (Dutheil et al., 2009).

**Figure 1:**
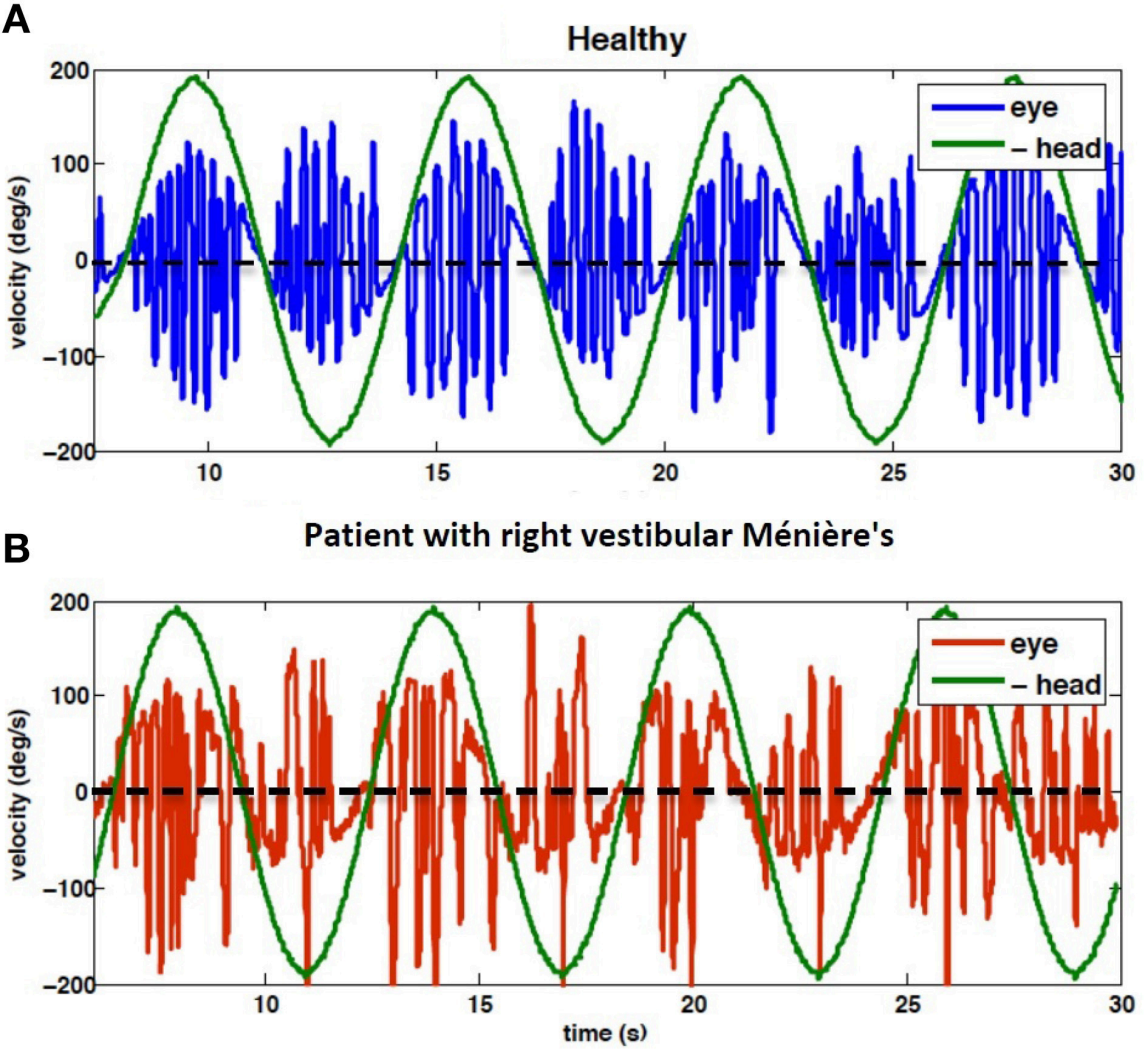
**Eye velocity during 1/6 Hz head rotation, peak velocity 200 deg/s; (A) VOR of control subject (***H***_**4**_), (B) VOR of subject diagnosed with right vestibular Ménière's disease (***P***_**9**_)**. Note that head velocity is inverted for better alignment of velocity traces. Positive head velocity values refer to right side rotation and vice versa. Clinical analysis focuses on the *envelope* of eye velocity segments that follow the same pattern as the head velocity, here harmonic. Thus, the rapid pulses associated with fast phases are ignored. The results of data analysis related to these recording are presented in Figures 3, 4.

Despite compensation, there remains some long lasting effects after losing vestibular afferent inputs, including deficits in the VOR gain especially during high acceleration head movements (Curthoys, 2000). As depicted in Figure 1, the VOR gain in patients is lower when rotating toward the lesion side, compared to controls, though the directional difference in nystagmus is attenuated after compensation.

Most reports on VOR compensation in unilateral patients focus on how the contra- vs. ipsi-lesion gain of the VOR varies in patients (Mantokoudis et al., 2014; Migliaccio and Schubert, 2014). If VOR dynamics are studied, traditional analysis methods are employed to study experimental data (Paige, 1989; Broussard et al., 1999; Maire and van Melle, 2000; Sadeghi et al., 2006). For example, a common approach is to remove fast phase intervals from the eye velocity profile during nystagmus and replace the missing data using interpolation, the so-called *envelope* approach. Such methods have been shown to produce biased estimates of the slow phase dynamics since they ignore the switching nature of the VOR system (Galiana, 1991; Ghoreyshi and Galiana, 2009) and the effect of initial conditions (Jalaleddini and Kearney, 2014) at the onset of each slow phase interval: each fast phase changes the initial point of the subsequent slow phase. New tools to analyze VOR nystagmus have been developed that allow accurate and reliable identification of its dynamics, despite switching (Ranjbaran and Galiana, 2014).

In this work, we re-examined the dynamic properties of the VOR in unilateral patients and controls using these new system identification techniques (described in Section 2.2). Our results suggest that asymmetry of the VOR responses during ipsi- and contra-lesion rotations is not limited to its gain- it is also significant in the time constant of the system. We explored how these asymmetries may be explained by modifications in the sensitivity and set-point of closed commissural loops between the VN. Simulations of a bilateral representation of the VOR replicate the observations of balanced resting point (no spontaneous nystagmus, and reduced gain asymmetry) at the cost of asymmetric time constants.

## 2. Materials and methods

### 2.1. VOR data from patient recordings

For the purpose of this study VOR data recorded for clinical evaluations from 20 patients with unilateral vestibular deficiency is used for analysis (8 Males, 12 Females, age range: 48 ± 14 years). In this group (*P*_1_−*P*_20_), 8 patients suffered from vestibular neuronitis, 7 were diagnosed with Ménière's disease and 5 had vestibular tumors. 12 patients had left side vestibular lesion and 8 had lesions on the right side. According to the clinician's diagnosis (AK) and the results of caloric tests, all patients had partial yet severe loss of vestibular functionality on the lesioned side. VOR tests on patients were performed 3 weeks or more after their last vertigo attack/operation/medication and they did not show any balance disturbance, such as spontaneous nystagmus. The same tests were performed on 10 controls (*H*_1_ − *H*_10_) with no known history of vestibular or ophthalmological disorders. Figure 1 shows an example of the VOR responses recorded from subjects *H*_4_ and *P*_9_.

The VOR data was recorded in the dark during 1/6 Hz sinusoidal rotation of peak velocity 200 deg/s using electro-oculography (EOG). Subjects were seated upright on a servo-controlled chair with their head fixed to the chair. Rotations were passive whole body around the earth vertical axis. For details of the experimental setup and calibration protocols, see Khojasteh and Galiana (2009). Data was recorded at 1 KHz, digitally low-pass filtered to 58 Hz and decimated to 250 Hz. Low pass filtering to 58 Hz is sufficient to reduce the effect of 60 Hz electrical noise in EOG signals while preserving the nystagmus bandwidth. Subjects signed a consent form describing the protocol, which was approved by the Institutional Review Board of McGill University, Faculty of Medicine.

### 2.2. Representation of VOR dynamics

**Figure 2:**
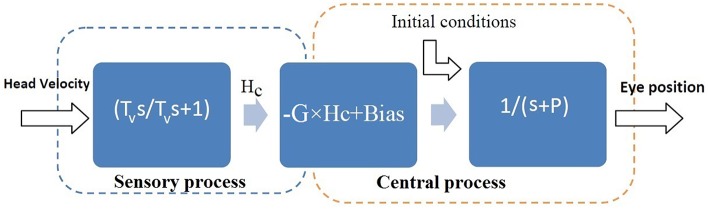
**Assumed structure for the slow phase VOR: linear dynamics followed by a Hammerstein system (Jalaleddini and Kearney, 2013)**. *H*_*v*_ is head velocity (deg/s), *H*_*c*_ is the sensory signal (spikes/s) and *E* is conjugate eye position (deg). *s* refers to Laplace variable and *T*_*v*_ refers to the sensory time constant in the first block. *G* is the steady state gain and *Bias* is added to model the bias in VOR responses due to asymmetries in the second block. *P* refers to the pole of the central processing in the third block and the central processing time constant is: $T=\frac{-1}{P},\;P<0$.

VOR data was first classified into slow and fast phases, as described in Ranjbaran et al. (2015). Here the study focuses on the dynamics of the VOR slow phases. The model formulation assumed for the slow phase system is described with a cascade of three blocks (Figure 2). The first block represents the sensory process which senses head velocity (*deg*∕*s*) and is modeled as a high-pass filter: $\frac{T_{v}s}{T_{v}s+1}$, where *s* refers to the Laplace variable and *T*_*v*_ is the vestibular time constant (Goldberg and Fernandez, 1971). The VOR, particularly in patients, often has a bias due to asymmetric resting rates in the neural circuitry as well as asymmetries in processing at each stage (sensor/brainstem). Hence the sensory description is expanded to include a bias term as depicted in the second block. The third block represents the VOR *neural integrator* in the brainstem, with low-pass first-order dynamics defined as $\frac{1}{s+P}$ and a time constant obtained from the pole, *P* as $T=\frac{-1}{P},\;P<0$.

We search for *T*_*v*_ in the range of 2–20 s (Goldberg and Fernandez, 1971; Raphan et al., 1979) and estimate the initial conditions and the unknown parameters (*G, P, Bias*) using the subspace identification method (details in Ranjbaran and Galiana, 2014). In order to estimate the dynamics for ipsi- and contra-lesion rotations separately, slow phase segments are grouped according to positive (rightward rotation) and negative (leftward rotation) head velocity values.

To evaluate the robustness of identified parameters in the VOR model in Figure 2, 95% confidence intervals of the estimated parameters are also computed using a Monte-Carlo approach. For this purpose, half of the slow phase segments are selected randomly and the model parameters are estimated. This is performed 200 times to obtain statistics of the estimated values. To evaluate/compare the significance of the estimated values in our analysis, standard *t*-tests are performed to test the null hypothesis and a *P*_*value*_ is computed.

## 3. Results

Identification of the VOR slow phase system as well as simulation studies are presented in this section.

### 3.1. Results from patient recordings

**Figure 3:**
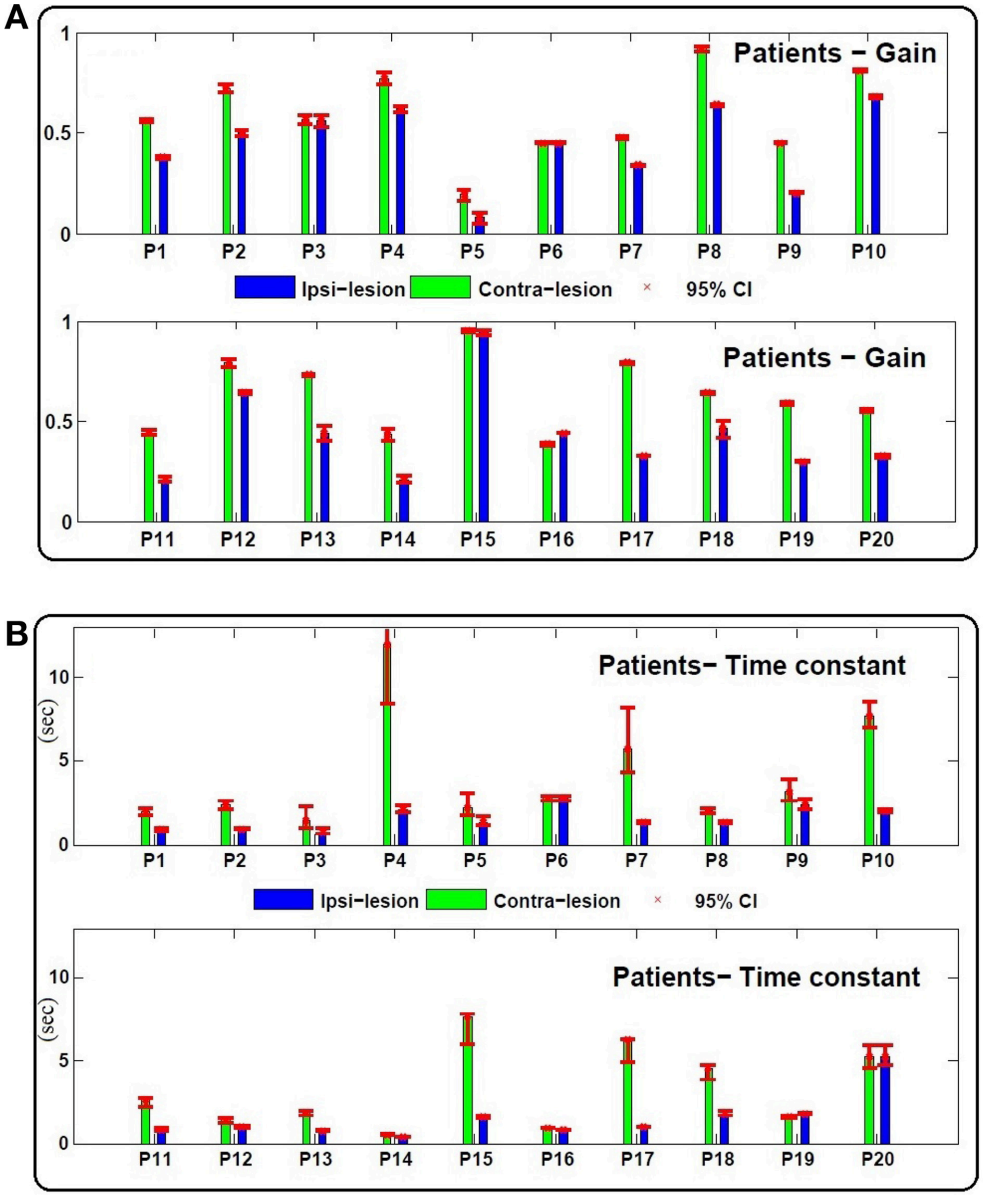
**(A)** Estimated gain (*g*_*co*_, *g*_*ip*_) and **(B)** central processing time constant (*T*_*co*_, *T*_*ip*_) values for contra and ipsi-lesion rotations in 20 unilateral vestibular patients (*P*_1_ − *P*_20_). Ninety-five percent confidence intervals of the estimated values are shown with red bars which are computed based a Monte Carlo study from 200 repetitions of parameter estimates from randomly selected data intervals in the record—each sample set had the same number of data points.

**Figure 4:**
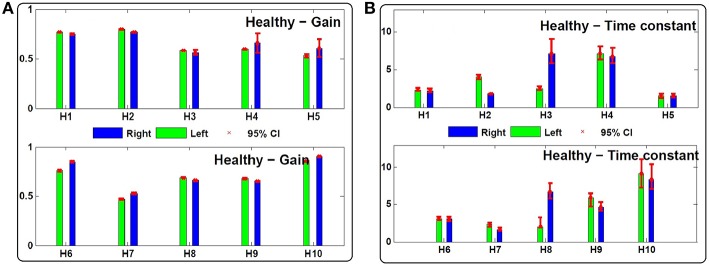
**(A)** Estimated gain (*g*_*l*_, *g*_*r*_) and **(B)** central processing time constant (*T*_*l*_, *T*_*r*_) values for leftward and rightward rotations in 10 control subjects (*H*_1_ − *H*_10_). Ninety-five percent confidence intervals of the estimated values are shown with red bars which are computed based on 200 Monte-Carlo studies as described in Section 2.2.

Figures 3, 4 depict the estimated values for the gain, *G*, and the central processing time constant, *T*, of the VOR slow phases during ipsi- (*g*_*ip*_, *T*_*ip*_) and contra-lesion (*g*_*co*_, *T*_*co*_) rotations in patients and right (*g*_*r*_, *T*_*r*_) vs. left (*g*_*l*_, *T*_*l*_) rotations in controls, respectively. Confidence intervals for the estimated parameters are shown with red bars to denote the robustness of the identification.

As expected, in most patients the gain during rotation toward the lesioned side is lower even after compensation: *g*_*ip*_ − *g*_*co*_ = −0.17 ± 0.12 < 0, (Figure 3). Two patients, *P*_3_ (left Ménière's) and *P*_6_ (left vestibular neuronitis) have near symmetric gains although at suboptimal levels. *P*_15_ (right vestibular neuronitis) on the other hand shows symmetric and a near optimal VOR gain. Asymmetric performance is not limited to the gain of the VOR. Interestingly, the time constant of the central process, *T*, also differs for ipsi- (*T*_*ip*_) and contra-lesion (*T*_*co*_) rotations. In most patients (except *P*_6_ and *P*_20_ with symmetric time constants), the estimated time constant of the brainstem circuit is smaller during ipsi-lesion rotations (*T*_*ip*_ − *T*_*co*_ = −2.14 *s* ±2.72 < 0).

Despite normal performance of the VOR in control subjects (no diagnosed vestibular condition or stated complaint), there can also be an asymmetry in the estimated gain and time constant of their VOR response: *g*_*r*_ − *g*_*l*_ = −0.02 ± 0.05 and *T*_*r*_ − *T*_*l*_ = −0.38 *s* ±2.33 (Figure 4).

A fair comparison between the extent of asymmetries in the response of patients and controls, however, should not depend on the rotation direction. Hence, we define two indices as the *relative* difference of the rightward vs. leftward rotation parameters:

(1) $$\text{Patients:}\{\begin{array}{l} G_{index}=\left|\frac{g_{ip}-g_{co}}{g_{ip}+g_{co}}\right|\\ T_{index}=\left|\frac{T_{ip}-T_{co}}{T_{ip}+T_{co}}\right| \end{array}\;\text{Controls:}\{\begin{array}{l} G_{index}=\left|\frac{g_{r}-g_{l}}{g_{r}+g_{l}}\right|\\ T_{index}=\left|\frac{T_{r}-T_{l}}{T_{r}+T_{l}}\right| \end{array}$$

**Figure 5:**
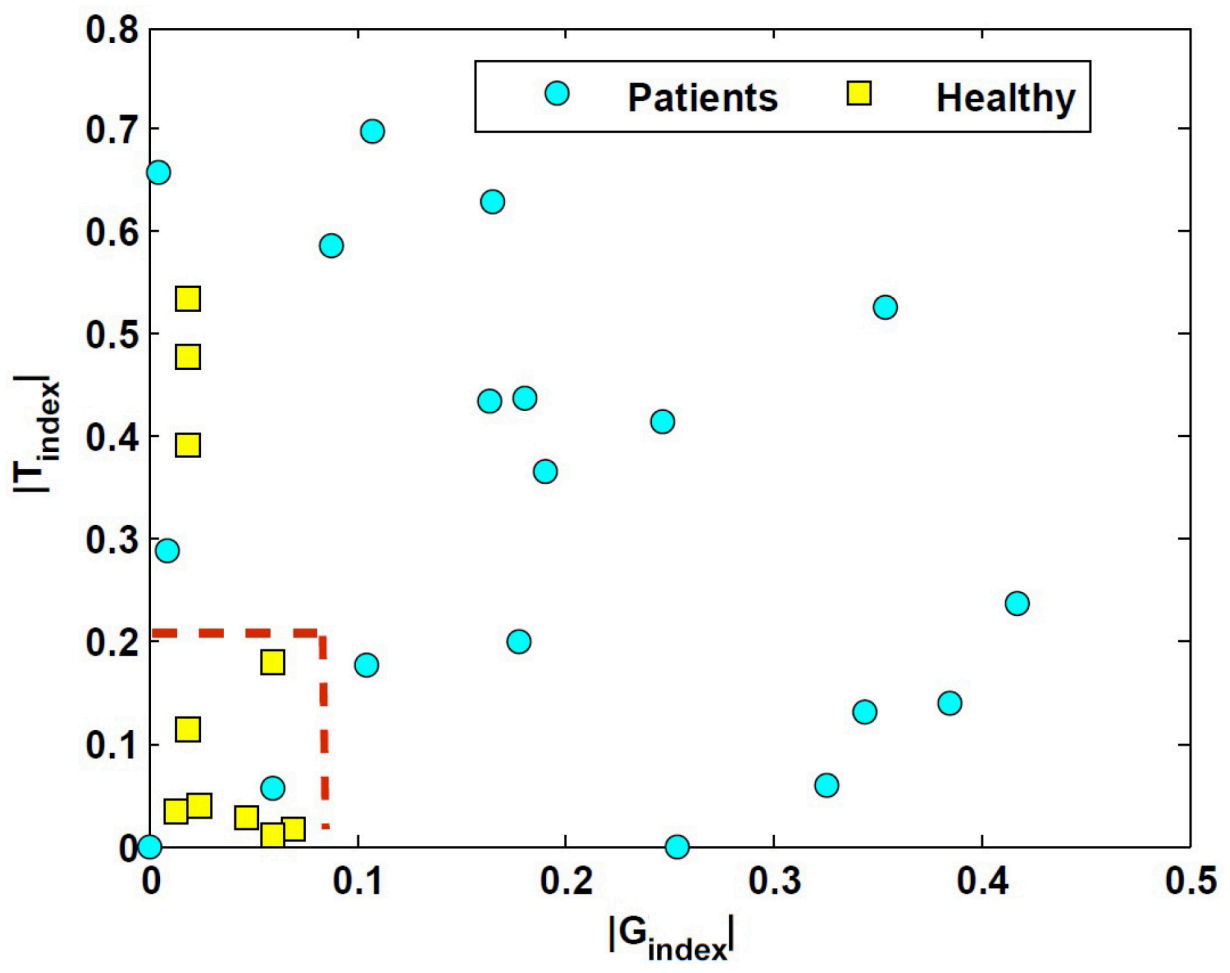
*****T***_***index***_ vs. ***G***_***index***_ in unilateral patients and normal subjects**. These indices refer to the relative difference of the estimated rightward-leftward parameters and emphasize the extent of asymmetries in the estimated VOR dynamics between the control and patient group.

These two indices normalize the extent of asymmetry. The *G*_*index*_ and *T*_*index*_ for both groups as well as their two-tailed *P*_*value*_ are computed in Table 1. The values suggest that the *degree* of asymmetries in both the gain and the time constant of patients is significantly larger than asymmetries in controls. Figure 5 shows this spatially by plotting the values of *T*_*index*_ vs. *G*_*index*_ for both the patient and control groups. While the control samples fall mainly close to the origin, reflecting small asymmetries, the markers for patients are widely distributed.

**Table 1:** **Symmetry levels in Control and Patient subjects for VOR gain and time constant**.

	**Patients**	**Controls**	***P*_*value*_**
*G*_*index*_	−0.19 ± 0.15	−0.02 ± 0.04	0.0009
*T*_*index*_	−0.33 ± 0.25	−0.33 ± 0.25	0.0425

*Data from Figures 3, 4 analyzed with Equation 1*.

In addition to the asymmetric properties of the responses, the average of the estimated gain and time constants between the two groups are also significantly different. As expected, the average gain in compensated patients, $\frac{g_{co}+g_{ip}}{2}=0.52\pm 0.19$, is significantly different and lower than the average gain in the control group, $\frac{g_{l}+g_{r}}{2}=-0.68\pm 0.12$ (*P*_*value*_ = 0.02). Interestingly, the average central processing time constant in patients $\frac{T_{co}+T_{ip}}{2}=2.62\pm 1.74$ is also significantly different and lower compared to the control group, $\frac{T_{l}+T_{r}}{2}=4.19\pm 2.32$ (*P*_*value*_ = 0.047). This suggests that on average the VOR responses in patients have faster dynamics compared to the controls, which reflects poor integration of the sensory information.

**Figure 6:**
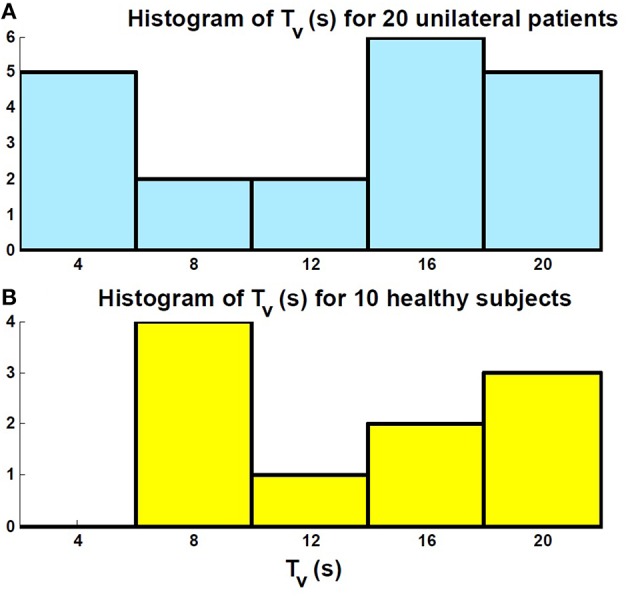
**Histogram of the estimated sensory time constant ***T***_***v***_ related to the first block in Figure 2**. **(A)** Patients; **(B)** Controls. There is no significant difference between the distribution of the estimated values in the two groups.

The histogram of the estimated time constant for the vestibular sensory process: *T*_*v*_, as depicted in the first block of Figure 2, is also shown in Figure 6 for both the patients and controls. It shows that the estimated vestibular time constant for both groups varies over a wide range (4–20 s). Thus, there is no significant difference in the estimated sensory time constant between the controls and patients after long-term vestibular compensation (*P*_*value*_ > 0.05).

### 3.2. Simulation results

In order to explain the results in Section 3.1, a simulation study was done. It is well established that the commissural network between the bilateral VN plays an important role in VOR compensation after vestibular lesion (Galiana et al., 1984; Graham and Dutia, 2001; Cullen and Minor, 2002). Here we tested how modulations in the resting activity of VN and in the sensitivity of commissural projections could affect the VOR dynamic properties after unilateral lesions.

**Figure 7:**
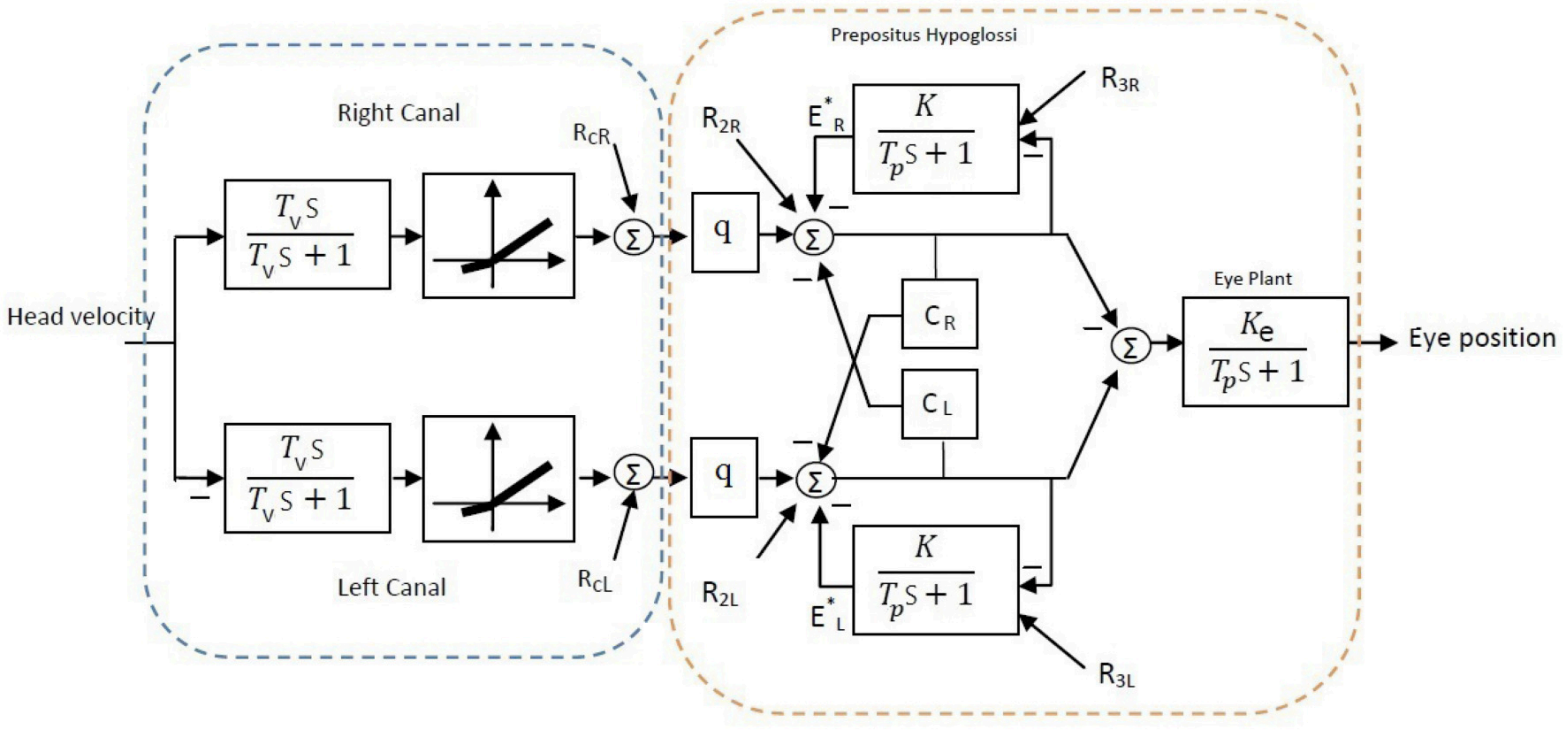
**Bilateral model of horizontal slow phase AVOR in the dark adapted from Smith and Galiana (1991)**. The model includes sensory component modeled with high-pass dynamics followed by a static nonlinearity. Eye plant and PH are modeled with first order low-pass dynamics. PH projects efferent copies of eye position $E_{R,L}^{*}$ while *C*_*R*_ and *C*_*L*_ refer to the commissural projection weights and *q* refers to the sensory projection weight. More details are provided in the text.

A simple bilateral slow phase VOR model was implemented in MATLAB Simulink (The MathWorks Inc., USA) as shown in Figure 7. This model was originally developed by Smith and Galiana (1991) and includes sensory dynamics as well as the central process of the VOR. The sensory stage includes high-pass dynamics with a time constant, *T*_*v*_, followed by an asymmetric nonlinearity around zero (gain for negative values: 0.4, positive values: 0.6) and a resting discharge for vestibular afferents, *R*_*cL, cR*_ (Goldberg and Fernandez, 1971). The central process includes the VN centers that received the sensory projections and the commissural projections, *C*_*R, L*_, as well as the efferent copies of monocular eye position provided by projections from the Prepositus Hypoglossi (PH). PH is modeled with low pass dynamics similar to the eye plant (time constant: *T*_*p*_, steady state gain: *K*_*e*_ for the eye plant and *K* for the PH). The model also allowed for resting discharges, *R*_2*L*, 2*R*_ and *R*_3*L*, 3*R*_ at the VN and PH centers, respectively. The model parameters were adapted from Smith and Galiana (1991) (Table 2). The VOR response generated by this slow phase model is valid for low amplitude sinusoidal rotations (no fast phase), or during brief head pulses, and is sufficient to illustrate the effects of circuit asymmetries. It would not be valid to run this model at the high head velocities seen in Figure 1 where the VOR response consists of mixed slow-fast intervals during nystagmus.

**Table 2:** **Numerical values of the model parameters**.

**Parameter**	***T*_*v*_**	***R*_*cR*_**	***R*_*cL*_**	***q***	***R*_2*R*_**	***R*_2*L*_**	***K***	***T*_*p*_**	***C*_*R*_**	***C*_*L*_**	***K*_*e*_**	***R*_3*R, L*_**
Intact	6 s	90 spk/s	90 spk/s	0.54	61 spk/s	61 spk/s	0.88	0.3	0.1	0.1	0.5	113 spk/s
Compensated	-	0	-	-	159 spk/s	-	-	-	0.01	0.6	-	-

“*-” are unchanged from intact case*.

As in the above experimental data analysis, virtual input-output data was first generated using the Simulink model and was then analyzed to estimate the apparent gain, *G*, bias and time constant *T* of the VOR central process shown in Figure 2. In the intact case with symmetric parameters on the two sides, the bilateral model reproduced symmetric VOR responses to head velocity input with zero bias. In this case *G* and *T* parameters can be defined theoretically as:

(2) $$\{\begin{array}{l} T=\frac{T_{p}(1-C_{R,L})}{1-K-C_{R,L}}=13.5\;s\\ G=\frac{qK_{e}}{(1-K-C_{R,L})T}=-1 \end{array}$$

With a vestibular lesion, defining *G* and *T* theoretically is not trivial since the model structure becomes asymmetric and nonlinear. Instead, we used system identification to study simulated VOR dynamics following lesion and then compensation, comparing rightward and leftward responses as for the experimental data.

**Figure 8:**
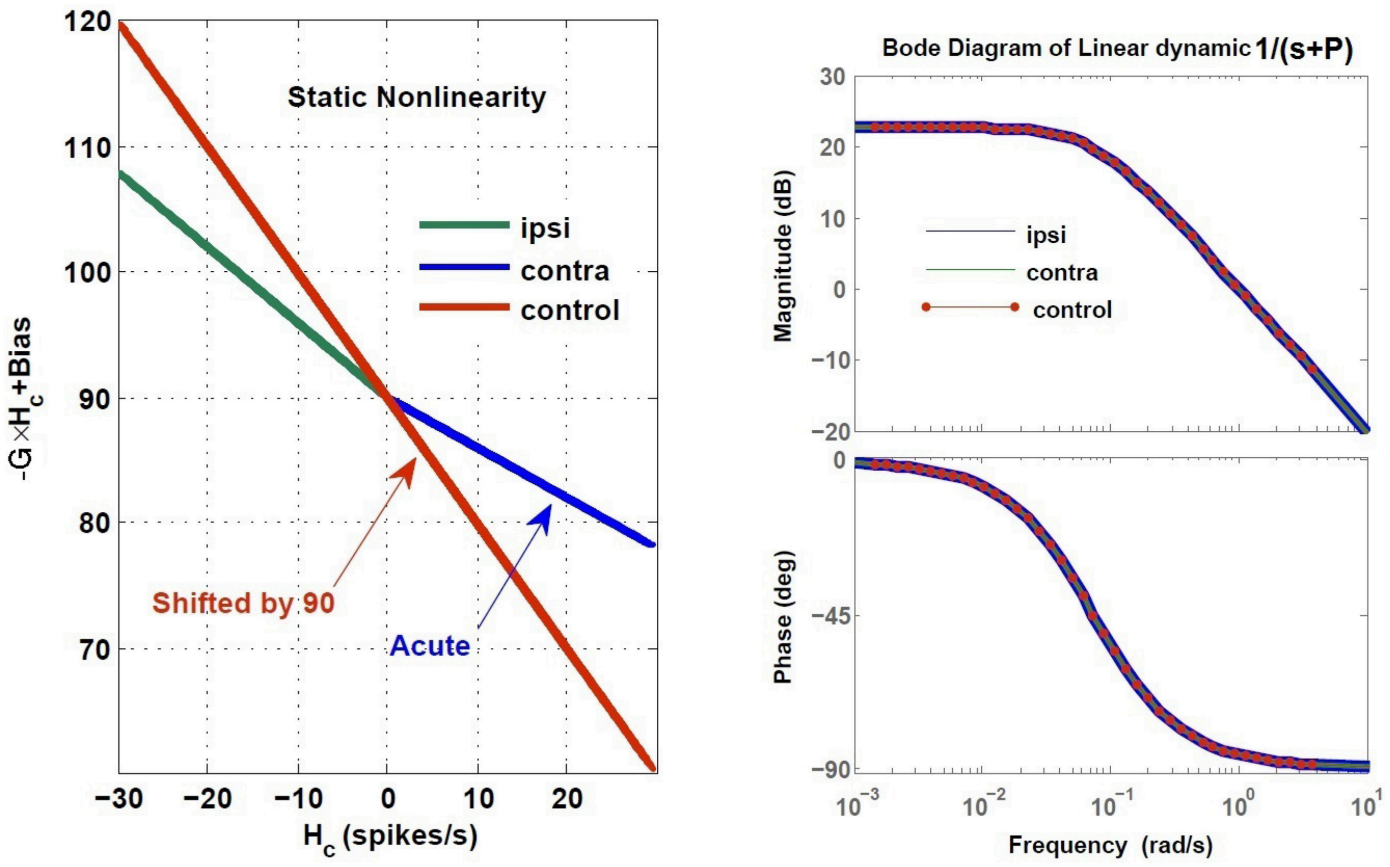
**Estimated dynamics of simulated VOR at acute stage of lesion with no compensation during ipsi- and contra-lesion rotations compared to intact VOR model**. Left panel provides the gain, *G* and bias of the central VOR process in the brainstem. Note that in the intact model, the bias in the VOR response is zero and the ideal gain is −1, thus the red line is shifted up by 90 (spikes/s), the vestibular resting rate *R*_*cR,cL*_, intentionally for comparison with the lesion case. The right panel shows the Bode plot of the expected form of VOR first order dynamic with time constant *T* = −1∕*P*, in the healthy condition.

Here, we assumed total loss of sensory projections including the afferent resting discharges (as in labyrinthectomy) from the right vestibular sensors. Figure 8 shows the estimated model (its gain curve: *G* and bias, and the Bode plot of the dynamics: $\frac{1}{s+P}$) from the simulated VOR at the acute stage; all other model parameters remained unchanged. It is seen that immediately following the loss of sensory input, the gain *G* is less than the normal case in both directions (−0.6 contra-lesion and −0.4 ipsi-lesion) and lowest for rotations toward the lesion side. Also a bias appears due to the asymmetry of the afferent resting discharges which is the main reason for initial spontaneous nystagmus in unilateral patients. Despite the asymmetry in the gain, there is no direction-dependent change in the estimated time constant (Bode plots in Figure 8) at the acute stage since the central processing as well as the commissural network remained unchanged and symmetric.

**Figure 9:**
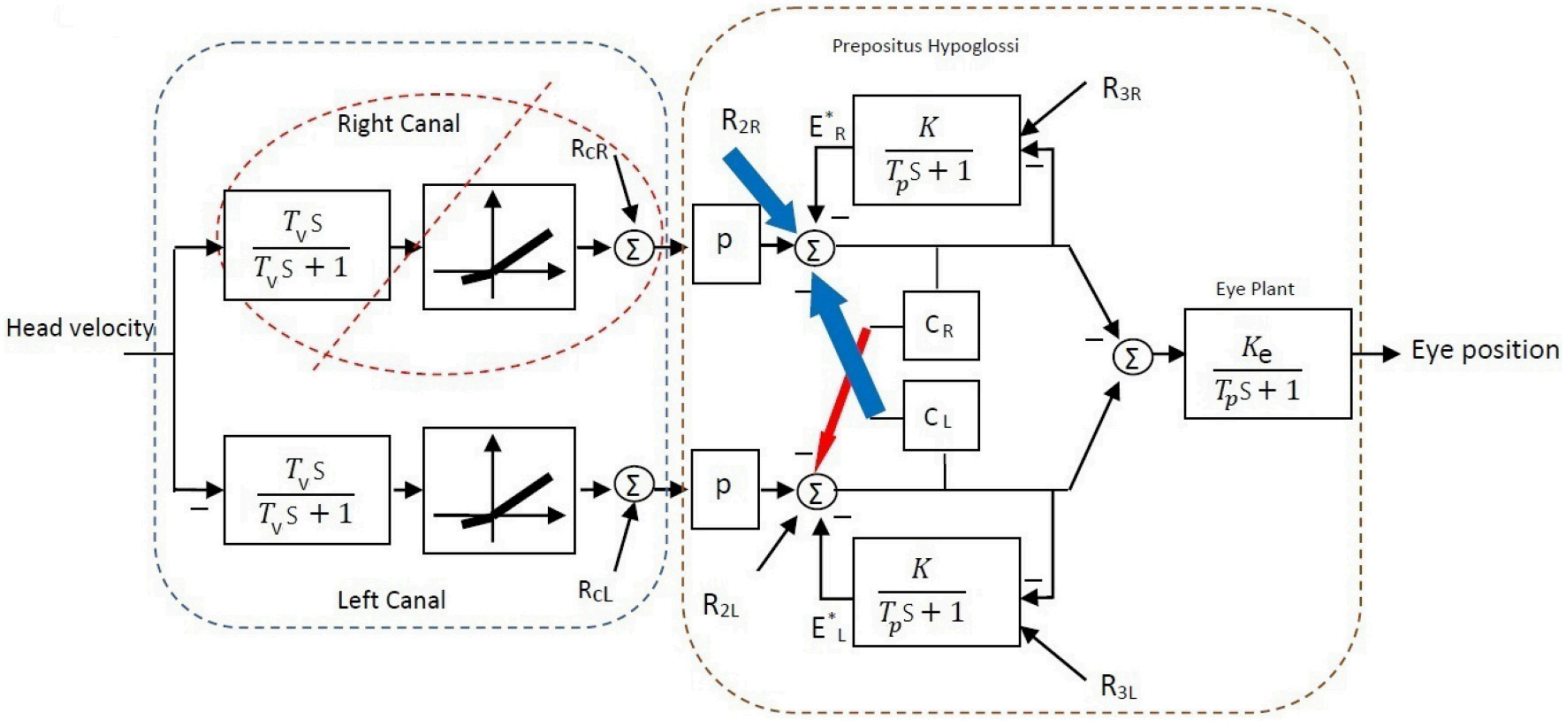
**Bilateral model of horizontal slow phase AVOR in the dark after unilateral lesion (right vestibular input) and compensation**. In order to balance gain and remove bias after unilateral lesion, the resting activity of ipsi-lesion VN (*R*_2*R*_) and commissural inhibition from the contra-lesion side (*C*_*L*_) are increased (blue arrows). Moreover, commissural inhibition from the ipsi-lesion side (*C*_*R*_) is decreased (red arrow). See Table 2 for parametric changes in the model.

**Figure 10:**
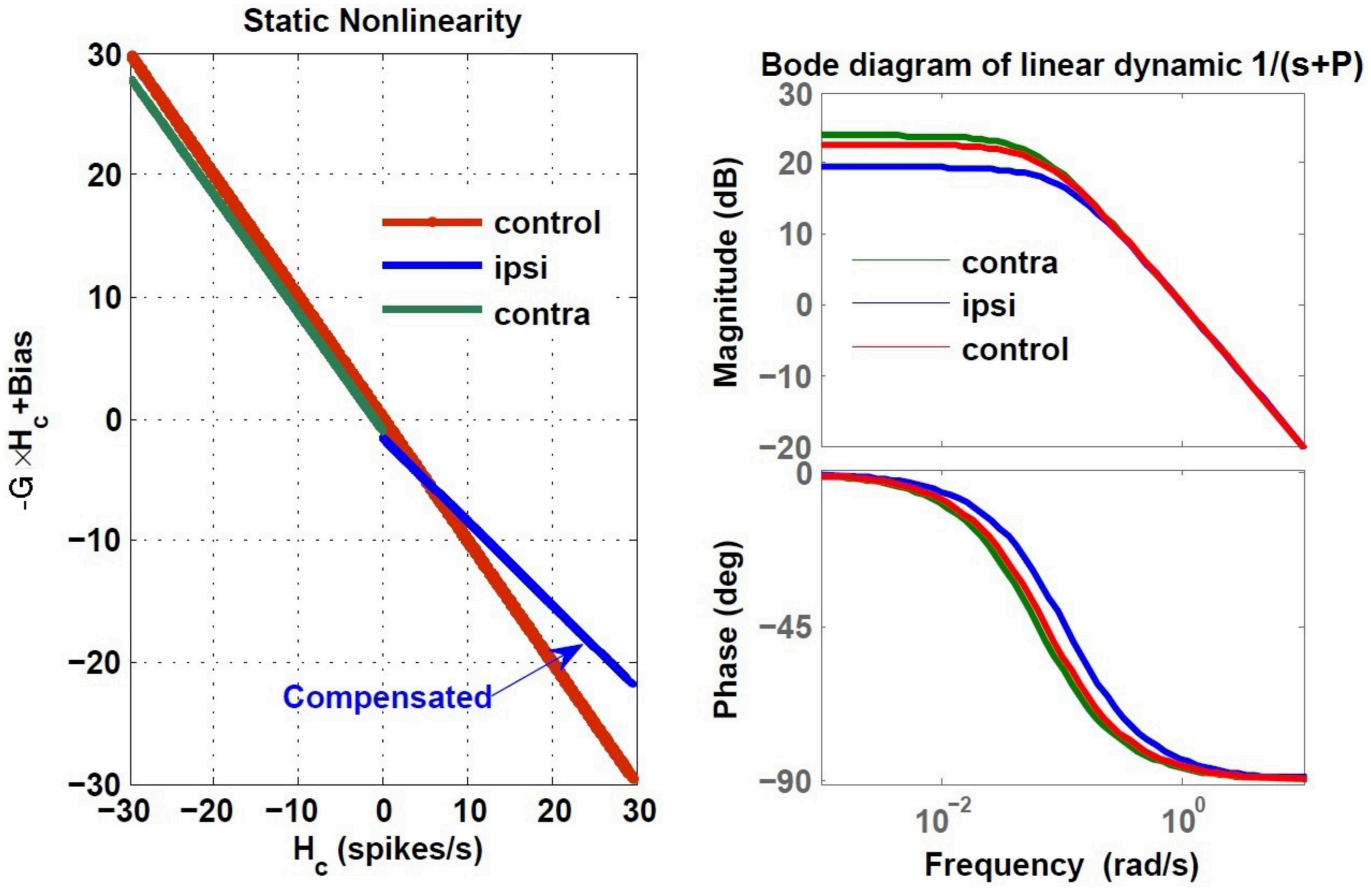
**Estimated dynamics of simulated VOR following compensation during ipsi- and contra-lesion rotations compared to intact VOR model**. Left block reflects the gain, *G* and bias of the central VOR process in the brainstem and the right block shows the Bode plot of the estimated VOR first order dynamic with time constant *T* = −1∕*P*. The ipsi-lesion dynamics (blue) imbed a smaller time constant than during contra-lesion rotations. The latter can remain close to the control case.

In order to restore VOR functionality after a unilateral vestibular lesion, as suggested in Cullen and Minor (2002), Graham and Dutia (2001), and Galiana et al. (1984), the resting discharge of the ipsi-lesion VN was increased to lower the bias across the midline. Moreover, the weight of commissural projections were also modified to increase the gain, with stronger inhibition from the contra-lesion side and less inhibition from the ipsi-lesion side. This is depicted graphically on the VOR bilateral model in Figure 9 and numerically in Table 2. The effect of such modifications in the model parameters on the overall response of the VOR is shown in Figure 10. It can be seen that the bias has almost disappeared while the gain, *G*, for rotations in both directions has increased compared to the acute stage (−0.96 contra-lesion and −0.68 ipsi-lesion); a lower gain toward the lesion side, despite the compensation persists as observed in experiments.

The compensation also affected the central time constant of the VOR during ipsi- and contra-lesion rotations. As it can be seen in the Bode plots in Figure 10, numerical changes to restore the VOR gain and null the bias in the model were associated with an asymmetry for the linear central dynamics. Consistent with our observation in VOR recordings, the time constant of the VOR during ipsi-lesion rotations (*T* = 9.37 *s*) was smaller than that during contra-lesion rotations (*T* = 15.62 *s*).

## 4. Discussion

It is known that different mechanisms are involved in VOR compensation. One possible site for restoring VOR functionality after lesions is the commissural network. In this work, we studied the dynamic properties of the VOR after compensation in patients with severe unilateral loss of vestibular functionality due to either vestibular neuronitis, Ménière's disease or tumors.

For the first time, a system identification technique was employed to accurately estimate VOR dynamics in patients during ipsi- and contra-lesion rotations that accounted for the effect of VOR switching on the dynamics. Firstly, it was seen that the range of estimated vestibular time constants (first block in Figure 2) in unilateral patients and controls varied over a wide range and there was no significant difference between the two groups (Figure 6). This suggests that, in fact, following vestibular compensation, the dynamics of the *effective* sensory projections to the central processing of the VOR are not significantly different in patients. Hence, assessment of the central dynamics might better localize the side of a lesion and the extent of compensation in patients.

Secondly, comparing the central dynamics of the VOR between controls and unilateral vestibular patients (second block in Figure 2) revealed that despite vestibular compensation, there remained significant asymmetry in the gain of the VOR, consistent with previous studies. Moreover, it was demonstrated that significant asymmetries in the time constant of the VOR with respect to the rotation direction are also present in patients- a novel indicator of possible vestibular deficits. In most unilateral patients, the system's time constant was smaller during ipsi-lesion rotations, the context with the lowest VOR gain. Finally the *average* of both the estimated gains and the central processing time constants in patients are significantly lower and different from the control group. In the presence of switching (nystagmus), a smaller central time constant can actually boost the apparent gain of averaged slow-phase segments. Hence modifying the VOR time constant here should be considered as a compensatory effect. However, it is not detectable with methods using envelope analysis- which instead estimate recovered symmetric VOR gains to mark a patient as “normal” or “compensated.” Our methods use identification from pooled slow phase segments and thus converge on true system dynamics, which are not normal.

Results of patient data analysis were then interpreted with model simulations. A simple bilateral model of slow phase VOR reproduced experimental observations in simulated VOR data. As expected, unbalanced sensory projections due to a lesion at the acute stage caused lower and asymmetric VOR gain and biased the VOR response (non-zero at null head rotation). Parametric adjustments of VN resting rates and the weight of projections in the commissural network restored the VOR gain to some extent and removed the bias. However, this resulted in asymmetry of the VOR dynamics during ipsi- and contra-lesion rotations, just as seen in the experimental data with our analysis. Hence, a reasonable hypothesis for biological VOR compensation is changes in resting rates at the VN level and modified projection strengths in the commissural system of the bilateral VN in both directions. These findings are compatible with prior neurophysiological observations (Dutia, 2010).

Faster VOR responses during ipsi-lesion rotations, i.e., smaller time constant, suggests poor integration of the primary signals on the lesioned side. However, it does have a beneficial effect on the traditional VOR envelope, by boosting slow phase speeds with eye deviations in the direction of the head rotation (nystagmus). Whether this integration deficiency recovers over time and to what extent, requires further studies. The extent of this dynamic asymmetry in the VOR responses can be used as an additional marker to evaluate the level of VOR compensation in patients and the effectiveness of any prescribed vestibular rehabilitation.

## Author contributions

MR performed the literature review, formulated the problem, analyzed the data, performed simulation studies, interpreted the results, drafted the manuscript and prepared the final version. AK provided clinical diagnoses for analysis validation, and proofread the final manuscript. HG provided overall supervision and advice on the problem formulation and solution, simulation and experimental studies and interpretation of the results as well as editing and proofreading the manuscript.

### Conflict of interest statement

The authors declare that the research was conducted in the absence of any commercial or financial relationships that could be construed as a potential conflict of interest.
